# Biomimetic Polyelectrolyte Complexes as Delivery Systems for Phytic Acid

**DOI:** 10.3390/biomimetics11100718

**Published:** 2026-10-09

**Authors:** Zoran Malbaša, Mihael Žvab, Anamarija Rogina

**Affiliations:** Faculty of Chemical Engineering and Technology, University of Zagreb, Trg Marka Marulića 19, HR-10000 Zagreb, Croatia

**Keywords:** chitosan, alginate, polyelectrolyte complex, phytic acid, films, antitumor properties

## Abstract

In this study, different chitosan–alginate polyelectrolyte complexes (PECs) were investigated as a biomimetic delivery system for phytic acid (PA), a natural antioxidant with potential anticancer activity. Physicochemical characterization, including ATR-FTIR, SEM/EDX, swelling analysis, and PA release profile, demonstrated that the polymer ratio strongly influenced film morphology, stability, and PA binding. Crosslinking generally produced smoother, more compact surfaces and resulted in reduced swelling capability, particularly in alginate-rich systems. PA release in normal saline varied significantly across formulations, with the 5:1 and 1:2 ratios releasing the highest amounts after seven days as a result of higher PA content. The stability tests exhibited strong ratio-dependent degradation, with the 1:1 films remaining the most stable across all tested media. Chitosan-rich films showed moderate mass loss (up to ~25%), whereas alginate-rich films, such as the 1:5 films, disintegrated due to excessive swelling and possibly weak interactions between polymer chains. PA-crosslinked samples exhibited similar trends; alginate-rich films lost up to 60–65% of their mass, while chitosan-rich samples demonstrated that a higher chitosan content produces more stable complexes, indicating a preferable interaction between PA and chitosan. Cytotoxicity assays using MG-63 osteosarcoma and HDFa fibroblast cells revealed dose-dependent effects. While PA-free materials did not affect the viability of either cell type, extracts of PA-crosslinked films at the highest concentration reduced the viability of MG-63 and HDFa cells. Importantly, moderate concentration of PA-crosslinked film extracts affected only MG-63 cells without harming fibroblasts, potentially suggesting mild preferential activity. Overall, the results demonstrate that PA-crosslinked PEC films provide a tunable, biomimetic platform with structural, physicochemical, and biological properties that can be modulated by adjusting the polymer ratio. The combination of enhanced stability, controlled PA release, and dose-dependent cytotoxicity offers potential for further development as antitumor materials.

## 1. Introduction

In the context of biomedical applications, biopolymers such as chitosan [1] and alginate [2] have been widely studied due to the polymer’s biocompatibility, tunable mechanical properties and structural similarity to native extracellular matrix (ECM) components. Chitosan is a natural cationic polysaccharide obtained by *N*-deacetylation of chitin and consists of randomly distributed 2-acetamido-2-deoxy-*β*-D-glucopyranose and 2-amino-2-deoxy-*β*-D-glucopyranose units. Chitosan’s linear charge density depends on the degree of deacetylation and the solution pH, with amino groups becoming protonated below pH ≈ 6.5. The degree of deacetylation determines the density of amino groups that can carry a charge, and the pH determines the degree of protonation of these amino groups. Alginate is a natural anionic polysaccharide extracted from brown algae, composed of *β*-D-mannuronic (M) and α-L-guluronic (G) acid residues arranged in M, G, and MG blocks. The relative amounts of the two uronic acid monomers and their sequential arrangement along the polymer chain vary widely, depending on the origin of the alginate. The functional groups of alginates are carboxyl groups, which carry a negative charge in aqueous media [3,4,5]. As oppositely charged polysaccharides in an aqueous solution, chitosan and alginate can interact and form a polyelectrolyte complex (PEC) structure upon mixing [6]. The interactions that contribute to PEC formation can include hydrogen bonding, van der Waals forces, and ionic bonds, depending on the chemical structure, the conformation of the polymer chains, and the conditions used to prepare the polymer mixture (nature of the solvent, pH, ionic strength, temperature, solution concentration, and mixing ratio). Polyelectrolyte complexes between chitosan and alginate are formed by combining complementary charges to generate self-assembled fibers or microgels, depending on the preparation method.

PECs have also attracted considerable interest as delivery systems because they facilitate the incorporation, stabilization, and controlled release of therapeutic agents [7,8,9,10,11,12,13]. The composition of PECs can be adjusted to modulate their swelling, degradation, and release behavior [7,11]. Consequently, the properties of PECs depend on the ratio of the precursors used to form the PECs owing to the different charges of the polymer chains [11]. Furthermore, polymeric systems can be designed for targeted delivery of therapeutics by responding to specific stimuli (e.g., pH or enzymes present) [1,5,14]. In the context of localized treatment of different tumors, PEC could be used as a potential delivery system, increasing local concentration of the therapeutic agent and reducing systemic toxicity [2,15,16]. More importantly, biomimetic design of delivery systems could also support tissue regeneration after therapeutic delivery. As ECM is formed by glycosaminoglycans containing negatively charged groups, the combination of chitosan and alginate could favor cell–biomaterial interactions [17].

Phytic acid (PA) is a natural source of phosphate, obtained from different types of seeds or grains and characterized by multiple reactive phosphate groups [18]. Phytic acid has a high negative charge density, which underlies its strong chelating capacity and notable antioxidant activity [19,20,21]. The phosphate groups, each bearing hydroxyl functionalities, enable phytic acid to interact with a wide range of ions and molecules, including metal cations, charged amino acids, and charged and uncharged regions of polysaccharides [22]. Through electrostatic interactions and hydrogen bonding, phytic acid forms stable complexes with chitosan and alginate [23,24,25]. Its highly phosphorylated structure resembles natural phosphate-rich molecules that stabilize biological tissues and mineralized matrices.

Recently, it was shown that phytic acid enhances osteogenic differentiation of bone marrow-derived mesenchymal stem cells (BMSCs) in a high-glucose environment [26]. As a natural antioxidant, phytic acid shows activity against breast and colon cancer [19,21,27]. Previously, Tantivejkul K. et al. [27] reported that phytic acid can interact with the chemotherapeutic drug doxorubicin, used in the treatment of various cancers [28,29], and can even achieve a synergistic effect when used with doxorubicin against breast cancer cells. However, standard chemotherapeutics such as doxorubicin, cisplatin, and methotrexate are given systemically, leading to non-specific distribution, severe side effects such as cardiotoxicity, and insufficient drug concentration at the tumor site [29,30,31,32]. Therefore, biomimetic chitosan–alginate PECs with incorporated phytic acid can be used as a potential therapeutic system in tumor treatment, avoiding the negative impact of conventional chemotherapy. Moreover, PECs in the form of films could be applied directly at the treatment site or as a coating on a supporting substrate where they function as a protective, therapeutic-releasing cover [33].

Hence, this research aimed to prepare biomimetic chitosan–alginate PEC films as potential delivery systems for phytic acid incorporated through electrostatic interactions. The physicochemical and biological properties of polyelectrolyte complex films prepared with different chitosan-to-alginate ratios and the impact of PA on the polyelectrolyte complex’s properties were investigated.

## 2. Materials and Methods

### 2.1. Materials

Chitosan (C; degree of deacetylation = 96.4%, viscosity = 293 mPa s; Chitoscience Chitosan 95/200, as declared by the manufacturer) was purchased from Heppe Medical Chitosan GmbH (Halle (Saale), Germany). Acetic acid (≥99.8%) was obtained from Lach-Ner (Neratovice, Czech Republic). Sodium alginate of low viscosity (A; viscosity = 4 mPa s as declared by the manufacturer) was purchased from Sigma-Aldrich (Saint Louis, MO, USA). Phytic acid (PA, 50% *w*/*w* in H_2_O; Sigma-Aldrich, St. Louis, MO, USA) was diluted to prepare a 1% (*w*/*w*) solution.

For the preparation of normal saline (NS, pH 7.4), sodium chloride was used. For the preparation of phosphate buffer solution (PB, pH 6.0), sodium phosphate dibasic heptahydrate and sodium phosphate monobasic monohydrate were used. For the preparation of phosphate-buffered saline (PBS, pH 7.4), sodium chloride, potassium chloride, disodium hydrogen phosphate, and potassium dihydrogen phosphate were used. All mentioned chemicals were purchased from Gram-mol, Zagreb, Croatia.

All reagents were of analytical grade and used as received, without further purification.

### 2.2. Preparation of Polyelectrolyte Films

Aqueous stock solutions (1% *w*/*w*) of sodium alginate and chitosan were prepared by dissolving sodium alginate in deionized water and chitosan in 1% *v*/*v* acetic acid, respectively. Polyelectrolyte complexes of chitosan and alginate (CA) were prepared at room temperature by mixing the solutions in five different volumetric ratios (Table 1) using sonication for 1 min at 40% amplitude (Bandelin Sonopuls 4200, Bandelin electronic GmbH & Co., KG, Berlin, Germany) using an SH 200G step horn. The final energy used was ~8.6 kJ for a 30 mL suspension. The maximum temperature measured at each suspension preparation was 37.4 ± 0.2 °C. The resulting suspensions were poured into aluminum plates (30 mL) and dried at 40 °C until dry films were obtained. Pure chitosan (CHT) and pure alginate (ALG) films were prepared using the same procedure.

### 2.3. CA Crosslinking with Phytic Acid

CA films intended for crosslinking were immersed in a 1 *w*/*w*% phytic acid solution, with a mass-to-volume ratio of 3.5 mg mL^−1^ and left for 24 h to crosslink. On the second day, the crosslinked films (CAPA) were rinsed with deionized water until a neutral pH was reached. Control films without PA (CA), were rinsed three times with deionized water. All samples were punched with an 8 mm diameter biopsy punch and dried at room temperature. Surface area and film thickness were measured after drying (*n* = 5). The CA films had an average area of 172.7 ± 8.9 mm^2^, while the CAPA films had an average area of 175.0 ± 10.0 mm^2^. The thickness of the CA films was 0.09 ± 0.01 mm, and the thickness of the CAPA films was 0.10 ± 0.01 mm.

### 2.4. Characterization of Polyelectrolyte Films

#### 2.4.1. ATR-FTIR

The compositional analysis of polyelectrolyte films was conducted using attenuated total reflectance Fourier transform infrared (ATR-FTIR) spectroscopy with a Shimadzu QATR-10 spectrometer (Shimadzu, Kyoto, Japan) equipped with a diamond crystal as the internal reflection element. Spectra were recorded in the wavenumber range 4000 to 400 cm^−1^ with a spectral resolution of 4 cm^−1^, and 32 scans were collected for each measurement. Measurements were performed at room temperature.

#### 2.4.2. Surface Morphology and Elemental Analysis

Scanning electron microscopy (SEM) was used to visualize the surface of the dry films, and the resulting micrographs provided insight into the morphological changes caused by different proportions of chitosan and alginate in the samples. The prepared scaffolds were coated with a thin layer of palladium and gold (SC7620 vaporizer, Quorum Technologies, Laughton, East Sussex, UK), and the analysis was performed using a scanning electron microscope (Tescan Vega III EasyProbe, Brno, Czech Republic) under high-vacuum conditions at an accelerating voltage of 10 kV. Energy-dispersive X-ray spectroscopy (EDX) (Quantax Compact, Bruker, Billerica, MA, USA) was also used for semi-quantitative measurement of phosphorus in the crosslinked samples without coating, in three randomly chosen surface areas per sample.

#### 2.4.3. Absorption Capacity

The absorption capacity of polyelectrolyte films was determined by immersing dry samples (*W*_D_) in phosphate-buffered saline (PBS, pH 7.4), normal saline (NS, pH 7.4), phosphate buffer (PB, pH 6.0), and demineralized water (H_2_O, pH 6.9) for 24, 48, and 72 h at 37 °C. After each time point, excess medium was removed by wiping along the edge of the well three times on both sides of the film, and swollen samples were carefully weighed (*W*_S_). Absorption capacity was calculated using Equation (1).

(1) Absorption capacity, % = ((Ws − W_D_)/W_D_) × 100

Measurements were performed in at least five replicates per system.

#### 2.4.4. Colorimetric Determination of Released Phytic Acid

The release of phytic acid from the individual samples was investigated at predetermined time points using the Megazyme Colorimetric assay kit (Megazyme, Bray, Co., Wicklow, Ireland). The results represent independent, interval-based measurements. The samples were weighed and incubated in normal saline (NS, pH 7.4) at 37 °C at a mass-to-volume (considered as material’s concentration) of 3.0 mg mL^−1^. The quantity of released phytic acid was measured at 1, 2, 3, and 7 days. Measurements were performed in at least three replicates at a wavelength of 655 nm using a microtiter plate reader (VICTOR Nivo Multimode Microplate Reader, PerkinElmer, Shelton, CT, USA).

#### 2.4.5. Cytotoxicity Assay

The cytotoxicity of non-crosslinked CA films and phytic acid-crosslinked polyelectrolyte films was evaluated using an indirect MTT ((3-(4,5-dimethylthiazol-2-yl)-2,5-diphenyltetrazolium bromide) assay on human osteosarcoma cells (MG-63, passage 4, Cytion GmbH, Eppelheim, Germany) and human adult dermal fibroblast cells (HDFa, passage 5, Thermo Fisher Scientific, Waltham, MA, USA). A free PA dose–response experiment was conducted on both cell types using 0.125, 0.250, 0.500, 0.750, 1.000, and 2.000 mmol L^−1^ solutions of PA prepared in complete cell culture medium, and IC_50_ was calculated by non-linear fit (inhibition vs. response, three-parameter model). The selectivity index (*SI*) was calculated as IC_50_(HDFa)/IC_50_(MG-63).

MG-63 and HDFa cells were cultured in high-glucose Dulbecco’s Modified Eagle Medium (DMEM, 4500 mg L^−1^ glucose; Capricorn Scientific GmbH, Ebsdorfergrund, Germany) supplemented with 10% fetal bovine serum (FBS, Sigma-Aldrich) and 1% penicillin/streptomycin (Sigma-Aldrich). When cells reached approximately 85% confluence, they were seeded into 96-well plates (Sarstedt) at a cell density of 5 × 10^3^ cells per 200 μL and allowed to adhere overnight in a humidified incubator (5% CO_2_, 37 °C).

Prior to testing, samples were sterilized under UV light for 30 min. CA samples were incubated in complete cell culture medium for 24 h at 37 °C at mass-to-volume ratios of 5 and 3 mg mL^−1^ (considered as the material’s concentration) while CAPA samples were incubated at 1, 3, and 5 mg mL^−1^. After incubation, extracts were collected. For cytotoxicity testing, 200 µL of each extract was added to cells seeded in 96-well plates and incubated for 24, 48, and 72 h.

At each time point, the testing medium was removed, and cells were washed with 1X PBS. Subsequently, 50 µL of MTT solution (0.5 mg mL^−1^ in complete medium) was added to each well and incubated for 4 h. After MTT incubation, 170 µL of dimethyl sulfoxide (DMSO) was added to dissolve the formazan crystals, followed by a 20 min incubation with constant shaking. Absorbance was measured at 570 nm using a microplate reader (VICTOR Nivo Multimode Microplate Reader, PerkinElmer, Shelton, CT, USA). Cell viability was calculated as a percentage relative to untreated cells. At cell viability below 70%, the material was considered cytotoxic according to ISO 10993-5:2009 [34].

#### 2.4.6. Statistical Analysis

The results are presented as mean values with standard deviation. Data comparisons were performed using two-way analysis of variance with polymer ratio (different chitosan–alginate ratios) as the row factor and medium (different test media) as the column factor (ANOVA), followed by the Tukey *post hoc* test. Significant differences between groups are indicated by asterisks, with the following thresholds: * *p* < 0.05, ** *p* < 0.01, *** *p* < 0.001, and **** *p* < 0.0001.

## 3. Results and Discussion

### 3.1. Film Preparation

After preparing the initial polymer stock solutions, the solutions were mixed thoroughly to create the polyelectrolyte system at different ratios (Table 1). The initial solutions were transparent. Upon mixing and during the sonication, microgels were formed i.e. a PEC suspension (Figure A1).

The stability of PECs is influenced by the nature and polarity of the polymers, their degree of polymerization, and the pH of the reaction medium. Looking at the properties of chitosan, its p*K*_a_ value is ~6.5, while the average p*K*_a_ value of alginate is ~3.38 to ~3.65, respectively [35,36]. Therefore, if the polyelectrolyte complex is in a solution with a low pH value, a large part of the amino groups of chitosan are positively charged (‒NH_3_^+^), while the carboxyl groups of alginate chains are in the form ‒COOH. As the pH value of the solution increases, the degree of ionized amino groups decreases, while the amount of negatively charged carboxyl groups of alginate increases. By changing the pH, the electrostatic interactions between the amino and carboxyl groups becoming stronger, forming a gel with a stable structure. When the same number of amino and carboxyl groups are positively and negatively charged, respectively, an equilibrium is established.

After dissolution, the pH of the alginate solution was approximately 6.5, and that of the chitosan solution was approximately 4.5. These pH values ensured that alginate was fully deprotonated and chitosan was fully protonated.

Upon mixing, the overall suspension maintained the pH of the chitosan solution (pH 4.5). At this pH, the chitosan chain contains the maximum number of positively charged amino groups, while the addition of dissolved alginate leads to partial protonation of the carboxyl groups, which can ultimately cause precipitation. At the moment of mixing, the amino groups of chitosan are linked to the carboxyl groups; however, if not all chains are interconnected, saturation of the carboxyl groups can still occur. When the pH drops to values close to the p*K*_a_ of uronic acids (typically reported in the range ~3.38 to ~3.65), a part of the carboxyl groups become protonated, reducing electrostatic repulsion and increasing the interchain association via hydrogen bonds [3,4]. As a result, viscosity increases, the chains compact, and precipitation or the formation of colloidal aggregates/gels may occur. In the work of Wasupalli G.K. et al. [37], it was shown that different fiber-like microgels are formed at different pH values during PEC preparation, which agrees with the microgels formed in this work (Figure A1). 

When immersing dry, non-crosslinked films in an acidic PA solution (pH value ~2), samples initially swell. At low pH, the proportion of free protonated amino groups of chitosan and the proportion of protonated carboxyl groups increase. Also, due to the negative charge of PA, the association of free amino groups of chitosan with PA occurs, favoring interactions between PA and chitosan with respect to the interactions between PA and alginate [31,38,39]. PA creates interactions through hydrogen bonds, ion–dipole interactions and electrostatic interactions and, in this way, can connect with alginate chains, creating a stable gel. However, it is important to take into account the ratio of PA and alginate, as shown in Nita L.E. [23]. The formation of stable PEC depends mostly on the C:A ratio, while the incorporation of PA can significantly impact the interactions between chitosan and alginate, especially at pH values lower/higher than that of PEC formation, leading to preferable PA–polymer interactions. Assumed interactions between PA, chitosan and alginate are illustrated in Figure 1. In the PEC structure, protonated amino groups on chitosan interact electrostatically with carboxyl groups on alginate, with additional stabilization provided by weaker hydrogen bonding. These interactions generate a relatively compact polyelectrolyte matrix. Upon PEC immersion into PA solution, the amino groups of chitosan and hydroxyl groups of alginate interact with phosphate and hydroxyl groups of phytic acid, forming an additional crosslink.

### 3.2. ATR-FTIR Spectroscopy

FTIR spectroscopy was used to investigate the interactions of phytic acid with PEC of different C:A ratios. In Figure 2, the spectrum of the chitosan film shows, between 3500 and 2980 cm^−1^, absorption bands correlated with overlapping stretching vibrations of O–H and N–H bonds. Absorption bands at 2921 and 2854 cm^−1^ correspond to asymmetric and symmetric stretching vibrations of the C–H bond. The absorption bands at 1637 and 1538 cm^−1^ correspond to the vibrations of amide I and amide II. The absorption band at 1320 cm^−1^ is related to the stretching vibration of the C–N bond, and the absorption band at 1259 cm^−1^ corresponds to the bending of the C–H bond in the CH_3_ group. The absorption band at 1151 cm^−1^ is attributed to C–O–C asymmetric stretching vibrations, and the absorption bands at 1063 and 1017 cm^−1^ are associated with C–O stretching vibrations characteristic of polysaccharides [11]. The spectrum shows an absorption band at 1403 cm^−1^ belonging to the COO^−^ group from acetate. When preparing the control sample by the casting method, the sample was not rinsed with demineralized water, so the resulting thin film is a chitosan acetate salt.

On the spectrum of the alginate film, between 3500 and 3000 cm^−1^, there is an absorption band associated with stretching vibrations of the O‒H bond in the hydroxyl group. At 2941 cm^−1^, an absorption band associated with stretching vibrations of the C‒H bond can be seen. Absorption bands appear at 1597 and 1406 cm^−1^ due to asymmetric and symmetric stretching vibrations of COO^−^ bonds. Absorption bands at 1087 and 950 cm^−1^ are associated with C‒O bond stretching vibrations and C‒O bond stretching.

The polyelectrolyte spectra show that changes in the chitosan-to-alginate ratio in the CA samples highlight the changes in characteristic bands of polymers (Figure 2). In the range between 3300 and 3000 cm^−1^, the absorption bands characteristic for hydroxyl groups of both polymers are expressed, regardless of the ratio. Absorption bands at 2922 and 2860 cm^−1^ correspond to asymmetric and symmetric stretching vibrations of the C‒H bond. More specifically, absorption bands associated with chitosan functional groups, amides I and II, at approximately 1641 and 1585 cm^−1^, become more defined with increasing chitosan content [6,11,40]. Increasing the alginate content results in more pronounced absorption bands that can be attributed to the asymmetric and symmetric stretching of the carboxyl groups of sodium alginate at 1593 and 1406 cm^−1^ [6,11,40]. At approximately 1070 and 940 cm^−1^, the C–O stretching is observed, depending on the chitosan-to-alginate ratio.

After crosslinking with phytic acid (Figure 2), it is evident that the individual bands attributed to amide I and amide II at 1635 and 1533 cm^−1^, as well as the carboxyl-related bands at approximately 1629 and 1531 cm^−1^, undergo noticeable spectral changes compared to the CA samples, as also shown in work of Nita L.E. et al. [23]. Furthermore, modifications likely arise from the potential binding of phytic acid to chitosan or alginate, depending on the polymer ratio, as shown by Stephy A. et al. [38]. After crosslinking with PA, characteristic alterations associated with the C–O stretching were observed at 1072 and 935 cm^−1^, as well as at approx. 1600 and 1530 cm^−1^.

Interestingly, another band appears at ~1730 cm^−1^, which is the most pronounced at the C:A ratio of 1:5. This band could be attributed to modified or saturated carboxyl groups. In the work of Kulig et al. [11], the band at approximately 1730 cm^−1^ possibly corresponds to the asymmetric stretching of –COO groups, indicating the formation of a complete polyelectrolyte complex with a higher proportion of alginate. More importantly, a new band was observed at ~500 cm^−1^, corresponding to O–P–O bending vibrations associated with phosphate groups in phytic acid [41]. Since the spectra of CAPA films show only changes in the position of characteristic bands, as well as overlapping of existing ones, electrostatic interactions between PEC and phytic acid are assumed [41,42].

### 3.3. Elemental Analysis

Since the PA is incorporated in the films by adsorption, the presence of phosphorus was determined by EDX analysis. The regions were randomly selected for elemental analysis, and the EDX results are shown in Figure 3. The CA films did not show any detectable phosphorus. The main elements present are carbon, oxygen, and residues from the transition metals from sample coating (which are excluded from the representation). However, CAPA films exhibited phosphorus, which also confirms that PA remains bound on the surface of the films after washing steps [5,41].

The phosphorus content in chitosan–alginate films crosslinked with phytic acid was estimated as atomic and mass percent (P, %) relative to the total detected elements. The results in Figure 3C show a clear decrease in phosphorus content with the reduction in chitosan content in the films. Samples with a 5:1 chitosan-to-alginate ratio contained approximately 8.0 ± 0.7 wt.%, followed by 2:1 with ~6.0 ± 0.2 wt.%, 1:1 with ~5.0 ± 0.2 wt.%, and 1:2 with ~4.5 ± 0.2 wt.%. The lowest value, 2.4 ± 0.1 wt.%, was observed for the 1:5 ratio. The results suggest that phosphorus incorporation depends on the chitosan fraction in the polyelectrolyte complex.

### 3.4. Morphology of the Polyelectrolyte Films

To examine the surface of the samples, electron microscopy was used. In Figure 4, films of CA polyelectrolyte complexes showed an irregular, fibrous surface. It was found that with an increase in the polymer ratio, more aggregates of polyanion–polycation complexes are formed, which is observed as increased irregularity in the structure. The differences in the surface of the films may be due to different ionic interactions dictated by the polycation-polyanion ratio [11,35].

CA film with a 1:1 ratio were grainy and had visible roughness, indicating saturated interactions between chitosan and alginate chains [35]. A more uniform and compact microstructure was observed for the 2:1 sample. With an increased fraction of chitosan, visible rough threads appear again. Sample 5:1 exhibited a rough and fibrous morphology compared to the 2:1 sample. The system with more alginate, 1:2, had a smoother surface compared to samples with excess chitosan, while at 1:5 the sample became inhomogeneous again. In conclusion, if chitosan or alginate dominates the ratio, samples tend to become inhomogeneous in surface texture.

After crosslinking with PA, the surface became smoother, except for the 5:1 ratio. This could indicate that films are affected by the preparation procedure, although they do not lose structural integrity after immersion in PA solution. A smoother surface may indicate swelling of the films, accompanied by potential dissolution and crosslinking in the PA solution. As a result, the polymer chains may rearrange and become more compact, as a result of PA crosslinking. The 5:1 sample retained string-like morphology after crosslinking.

Similar observations of the microstructure of films based on sodium alginate and chitosan were found in previous studies [11,35,43].

### 3.5. Absorption Capacity in Different Media

Absorption capacity governs fluid uptake, swelling, mechanical softening, diffusion rates of solutes, and the film’s ability to maintain contact with tissues or substrates. Polysaccharide films interact with their environment, and those interactions depend strongly on the medium’s composition. Measuring absorption capacity in multiple media may suggest how the film will behave in real applications. Different pH values can affect the protonation and deprotonation of functional groups in chitosan and alginate, which dictate the uptake of the medium. In addition, the presence of different ions and ionic strength of the solution can affect medium absorption. Salts can screen electrostatic interactions and can tighten or expand the polymer network, while multivalent ions can form new crosslinks or compete with existing ones [44]. For these reasons, different media were used to investigate the swelling of CA and CAPA films.

Both CA and CAPA were immersed in dH_2_O, which serves as a baseline: no additional ions are present, the pH is near neutral, and only water molecules can interact with the polymers. The next medium used was normal saline, which has the ionic strength similar to extracellular fluid and can screen charges since it contains sodium and chloride ions. Furthermore, phosphate-buffered saline was used to determine absorption capacity at physiological pH and, to examine how CAPA samples respond to the presence of phosphate. Finally, phosphate buffer at pH 6.0 was used as a buffer without physiological salts. PB can serve to assess swelling at a pH of 6.0, which is characteristic of the tumor microenvironment.

Figure 5A shows the swelling behavior of control and crosslinked films in different media after 72 h of exposure, with comparable trends after 24 and 48 h (Figure A2), indicating the system reached equilibrium swelling.

According to the swelling results in dH_2_O, sample CA at a 1:1 ratio absorbs the least (less than 100%), likely due to strong ionic interactions between amino and carboxyl groups. Generally, with an increase in the amount of chitosan, swelling slightly increases: the 2:1 sample absorbs more (~150%), while the 5:1 sample absorbs the most (up to ~200%). By increasing the proportion of alginate, the swelling of CA notably increases. The 1:2 sample swells to around 300%, while the 1:5 sample dissolves in dH_2_O. In natural saline, the 1:1 sample absorbs the least, around 100%, while the 2:1 and 5:1 samples swell similarly, around 150%. With an increase in alginate, the 1:2 sample swells around 150%, and the 1:5 sample absorbs around 400%. All the samples remained integrity after the 72 h of immersion in normal saline. In PBS, the 1:1 sample absorbs above ~100%. The 2:1 and 5:1 samples absorb ~120%. Alginate-rich samples absorb significantly: 1:2 absorbs 350%, and 1:5 absorbs 1500%. In PB, the 1:1 sample absorbs ~70%, and with an increase in chitosan, the 2:1 sample absorbs around 100%. The 5:1 sample absorbs ~110%. With an increase in alginate, the 1:2 sample absorbs 150%, and the 1:5 sample absorbs 800%.

When chitosan and alginate are combined in the same ratio, the charge ratio of positive and negative ions is nearly equal, forming the most stable type of polyelectrolyte complex. Changing the charge ratio alters the conformation of the polyelectrolyte complex. As the positive charge increases, chitosan becomes dominant in the outer part of the core–shell structure, while the inner part becomes alginate, and vice versa [42]. Chitosan-rich films (5:1, 2:1) form stronger networks with lower swelling because the abundance of positively charged amino groups creates strong ionic crosslinks. Balanced ratios (1:1) show minimal swelling due to optimal charge pairing. Alginate-rich films (1:2, 1:5) swell dramatically at a neutral pH due to increased negative charge density and structural features such as internal “pockets” that trap water and amplify swelling [43].

According to the obtained results, the swelling capacity depends on the pH of the medium and the ions to which the samples are exposed. Polyelectrolyte complexes formed by chitosan and anionic polymers in the pH range p*K*_a_ (anionic polymer, alginate) < pH < p*K*_a_ (cationic polymer, chitosan) show the lowest swelling. At a pH > 6.5, most of the amino groups of chitosan become ‒NH_2_, and the carboxyl groups of anionic polymer are ionized; therefore, the repulsive forces between the carboxyl groups should lead to an increase in swelling [5,44]. The amount of each polymer should also be taken into account, since the mentioned pH range is defined for a stoichiometrically equal proportion of chitosan and alginate. In addition to the observed pH values of 7.4 and 6.0, a possible influence is expected from the foreign ions, i.e., ionic strength [45]. Namely, for the CA 1:1 sample, the highest capacity is achieved in PBS, which is expected, while the values are slightly lower in normal saline, PB and water. At pH 6.0, the greatest contribution to swelling is made by carboxyl groups, with a relatively small proportion of amino groups. At elevated pH, chitosan swells the least, and the swelling of the carboxyl groups of alginate prevails. Normal saline contains only sodium and chloride ions. Alginate prefers binding with sodium ions, just as positively charged amino groups prefer interaction with free chloride ions, which can lead to altered swelling. This often results in moderate swelling, but lower swelling than in more complex salt solutions. On the other hand, PBS is a solution of several different salts (multiple ions such as Na^+^, K^+^, and phosphate species) that can bind to a single polymer chain. Salt precipitation can also occur, as a result of which swelling can be more pronounced. Multivalent phosphate ions can bind to several sites on the polymer chains, altering the network structure, while salt precipitation within pores increases osmotic pressure and drives additional water uptake. In contrast, demineralized water lacks ions to shield repulsive forces, allowing alginate chains to fully hydrate. This leads to extreme swelling or even dissolution of alginate-rich samples, as observed by disintigration of alginate-rich films after prolonged immersion. 

After crosslinking with phytic acid, chitosan-rich films and C:A 1:1 showed similar swelling as before crosslinking, while significant changes were detected for alginate-rich films. The 1:1 CAPA film consistently showed the lowest equilibrium swelling in every medium tested. A higher chitosan fraction produced progressively higher water uptake: the 5:1 films absorbed more than the 2:1 films, and both absorbed more than the 1:1 formulation. Increasing the alginate fraction also increased swelling relative to the 1:1 sample, with a higher absorption capacity than samples with more chitosan. But alginate-rich films swelled less than the uncrosslinked CA film in dH_2_O, PBS and NS. 

Based on the amount of absorbed medium in each film, it is evident that in water the 1:1 ratio absorbed about 75%, the 2:1 ratio about 80%, and the 5:1 ratio over 90%. All samples retained structural integrity, indicating that they became more stable after crosslinking compared to the pure CA film. With increased alginate content, the 1:2 ratio absorbed around 80% of the water and the 1:5 ratio about 150% of the water. In natural saline, the 1:1 ratio absorbed around 80%, the 2:1 ratio around 100%, and the 5:1 ratio about 125%. Samples with more alginate, the 1:2 ratio absorbed approximately 125% and the 1:5 ratio 250%, showing considerably lower absorption capacity compared to CA films under the same conditions. Interestingly, in PBS some samples swell similarly to CA films. The 1:1 samples absorb similarly to CA, around 100%, but with an increased chitosan fraction, the absorption capacity remains almost unchanged. The 2:1 and 5:1 CAPA samples absorbed more in PBS than the CA films, reaching 130% and 150%, respectively. In contrast, samples with more alginate absorbed less in PBS than CA films, with the 1:2 ratio absorbing around 300% and the 1:5 ratio around 800%. Finally, the highest amount of absorbed medium was observed in PB. The CAPA samples with more chitosan absorbed similarly to CA films at the same ratios. The CAPA 1:1 absorbed slightly more than CA films, around 100%, while the 2:1 films absorbed more than 100%, and the 5:1 films absorbed almost 150%. Alginate-rich films absorbed less than CA films at the same ratios: the 1:2 ratio absorbed around 650%, and the 1:5 ratio absorbed around 900%.

The release of phytic acid obviously affects swelling of alginate-rich films in PBS and PB. Phytic acid itself can form chelates with ions present, such as sodium or potassium, and may result in higher water uptake [46]. Furthermore, as both PB and PBS contain phosphate salts, the influence of free phosphate groups may explain the difference in absorption capacity, but further investigation is needed.

### 3.6. Stability of the Polyelectrolyte Films

After the swelling capacity was investigated, the films were rinsed with distilled water and dried to constant mass to determine the residual mass of the samples. Figure 5B shows that the CA 1:1 ratio was the most stable across all solutions, with negligible mass change. The CA 1:5 ratio dissolved during immersion in water after 72 h, while in PBS and PB samples, mass decreased to 75% of the original, indicating loss. Due to the higher amount of alginate, these samples swell more, allowing deeper penetration of solvents, especially at a neutral or slightly lower pH, such as 6.0. Moreover, the mass loss may indicate that the interactions at this ratio are weak because the polymer chains prefer to form stronger interactions with ions within the solutions. Interestingly, in normal saline, CA samples with higher chitosan or alginate fractions showed greater mass loss than the 1:1 sample.

Samples crosslinked with PA exhibited different behavior. Across all the solutions investigated, the most significant change happened in PBS and PB. The 1:5 ratio loses more than 60 to 65% of the total mass, which could indicate the dissolution of the samples. The 1:1 ratio exhibited a similar mass loss of ~25% as the 5:1 and 2:1 indicating that more chitosan can provide a more stable network by PA crosslinking. In PBS and PB, pH was not affected (Figure 5C).

In demineralized water, all crosslinked samples lost a few percent in mass, approximately 5 to 10%, depending on the ratio. This mass loss can be substantiated by the pH change when phytic acid was released from the samples (Figure 5C). Similarly, the pH decrease was observed in NS due to PA release, which could affect the stability of polyelectrolyte films.

A possible reason why alginate-rich films lose a lot of mass could be delamination of the films after crosslinking with PA (Figure A3). Phytic acid can saturate the carboxyl groups of alginate chains. Assuming that phytic acid forms stronger interactions with chitosan amino groups, a loss of stability may occur. If such a system is additionally exposed to metal ions or phosphates from PBS or PB, the non-crosslinked alginate chains may dissolve.

### 3.7. Release of Phytic Acid from CAPA

The mass of phytic acid released per sample was determined in natural saline using a colorimetric method. Briefly, the method is based on extracting phytic acid from solid matrices (digestion in hydrochloric acid followed by neutralization with sodium hydroxide) and then enzymatically hydrolyzing it to convert phytic acid into measurable phosphorus. Because the polysaccharide films are not soluble in hydrochloric acid, the option was to measure the released phytic acid in a sodium chloride solution, which contains the same amount of sodium and chloride ions as the solution expected in the assay itself.

Figure 6 generally shows that more phytic acid was detected with longer incubation time. After seven days, the highest amounts of phytic acid per sample’s weight, 78 µg mg^−1^ and 64 µg mg^−1^, were measured for samples 5:1 and 1:2, respectively. The lowest amount was detected for sample 1:5 at around 40 µg mg^−1^. For CAPA 2:1, the PA quantity measured was 46 µg mg^−1^, while for 1:1 it was 57 µg mg^−1^.

The release of PA is dictated by the swelling properties (i.e., ion presence). According to the swelling results, the PA release profile follows the swelling trend in normal saline for three days. Furthermore, samples were stable and had lost 5 to 10% of their original mass. The highest and the lowest PA release for CAPA 5:1 and 1:5, respectively, correlate with the amount of PA incorporated into CAPA as estimated by semi-quantitative EDX analysis. Certainly, the conformation of PEC should also be considered when changing the C:A ratio, as suggested by Saether H.V. et al. [4]. When positive or negative charge is increased, the excess charged phase moves to the outer surface layers of the polyelectrolyte complex. Furthermore, the higher PA quantity released in normal saline over longer periods indicates the weakening of interactions between PA and PEC due to the presence of foreign ions.

Slight variations in PA release profile in the 2:1, 1:1 and 1:2 systems could be a result of different conformations and interactions in the presence of salt. Hence, additional analytical methods to determine PA release are needed to fully investigate the correlation between PA binding and release at different CA ratios of CAPA.

### 3.8. Cytotoxicity

Cytotoxicity of the samples was investigated indirectly by MTT assay, measuring cell metabolic activity. Extracts of the non-crosslinked and crosslinked samples were used to treat MG-63 osteosarcoma and HDFa fibroblast cells. Non-crosslinked chitosan–alginate films did not negatively affect the viability of MG-63 or HDFa cells after 72 h at all concentrations tested (Figure A4), indicating non-cytotoxicity, consistent with the literature [47]. The introduction of phytic acid made significant changes in cell viability, as shown in Figure 7. At the highest and moderate concentrations of CAPA films (5 and 3 mg mL^−1^), the samples reduced the viability of the MG-63 cells compared to the negative control, particularly for chitosan-rich samples. At the lowest tested concentration, chitosan-rich samples were at the threshold of cytotoxicity, while all other systems were not cytotoxic to osteosarcoma cells. Regarding the effect of materials on HDFa cells, it is evident that at the highest material concentration, samples 5:1 and 1:2 reduced the cell viability relative to the negative control. Other samples were at the threshold of cytotoxicity. At a moderate concentration, the samples did not affect cell viability after 72 h of incubation; moreover, an increase in cell viability compared to the negative control was observed in 2:1, 1:1, and 1:5 samples. At the lowest concentration, all samples were non-cytotoxic.

Similar results of PA impact on different cell types have been reported by Saverino A. et al. [19], showing antitumor activity, inhibiting proliferation and inducing apoptosis in malignant cells while causing minimal cytotoxicity toward healthy cells. Consistent with the cited work, a preferential activity of PA towards cancer cells may be explained by disruption of iron-dependent metabolism and ROS-sensitive survival pathways [48]. A similar observation of the impact of PA on tumor cells was reported in research conducted by Ju Q. et al. [49]. Investigating the effect of manganese nanoparticles crosslinked with PA, they observed that after 24 h, there was a drastic decrease in the viability of human osteosarcoma cells (U2OS and 143B) due to prolonged exposure to PA-modified nanoparticles. Likewise, treatment with PA solutions alone for 24 h at concentrations of 100, 125, and 150 µg mL^−1^ resulted in reduced cell viability. Another study [50] investigated the impact of phytic acid on human periodontal ligament fibroblasts (HPDL). They observed that at three concentrations (0.005%, 0.01%, and 0.02%), phytic acid had a positive effect on the viability of HPDL fibroblasts at optimal glucose levels, indicating that PA does not affect healthy fibroblasts. More importantly, a similar result was reported by Ghilan A. et al. [24], who investigated combinations of different ratios of PA and carboxymethyl chitosan. At the highest concentration of 0.5 mg mL^−1^, the extract of the samples with the highest amount of PA (chitosan-to-PA ratio of 6:1) was not cytotoxic to human dermal fibroblasts after 24 h.

To support the conclusion on PA cytotoxicity, a PA dose–response assay was performed on MG-63 and HDFa cells (Figure A5). The resulting viability profile indicates a gradual reduction in viability of both cell types. Since the true IC_50_ values would be in a higher PA concentration range, IC_50_ was estimated; for HDFa cells it was 4.585, while for MG-63 cells it was 3.727. Additionally, the estimated selectivity index value was 1.23, suggesting only mild preferential activity. Certainly, true selectivity cannot be confirmed at this stage. To determine whether PA genuinely exhibits selective cytotoxicity, further evaluations across multiple osteosarcoma subtypes and corresponding healthy cells are required.

Considering the acidity of PA, pH values of the investigated extract were measured. The CA extracts did not influence the pH of cell culture media, regardless of the chitosan-to-alginate ratio, reaching an average pH of 7.50 ± 0.06 after 24 h of material immersion. The average pH of CAPA extracts was slightly lowered and depended on the extract concentration: 7.20 ± 0.10 for 5 mg mL^−1^, 7.22 ± 0.08 for 3 mg mL^−1,^ and 7.34 ± 0.07 for 3 mg mL^−1^. Specifically, the 5:1 and 1:2 ratios lowered the pH value the most, which correlates with the PA release experiment. Nonetheless, the PA released did not disrupt the physiological pH of the cell culture media.

Overall, cytotoxicity assays of CAPA films indicate an antitumor potential that need to be further investigated in a more complex tumor microenvironment.

## 4. Conclusions

Chitosan–alginate polyelectrolyte complex films crosslinked with phytic acid, in five different chitosan-to-alginate ratios, were developed and evaluated for biomedical applications as biomimetic films. SEM analysis revealed that CA films exhibited irregular, fibrous surface morphologies whose roughness depended strongly on the chitosan/alginate ratio, indicating stoichiometry-dependent ionic interactions within the PEC network. Crosslinking with phytic acid generally produced smoother, more compact surfaces. EDX confirmed the presence of phosphorus exclusively in CAPA films, demonstrating that PA remained within polysaccharide matrix after rinsing. FTIR spectra further indicated electrostatic binding of PA to chitosan and alginate chains by a slight shift in absorption bands associated with amino, amide, and carboxyl groups and by the appearance of characteristic phosphate bands. Swelling capacity showed that CA and CAPA films were responsive to pH and ionic strength. CA films, particularly those rich in alginate (1:2 and 1:5), exhibited high swelling in neutral and slightly basic media due to increased alginate ionization and reduced chitosan protonation. In contrast, CAPA films showed significantly reduced swelling, demonstrating the crosslinking effect of PA. PB induced the highest swelling in alginate-rich samples of CAPA, while NaCl and PBS produced moderate swelling. In demineralized water, alginate-rich CA films showed extreme swelling and even dissolution, highlighting the destabilizing effect of ion-free environments on alginate-dominated networks. Residual mass measurements indicated that CA films with balanced ratios (1:1) were the most stable, whereas alginate-rich films lost substantial mass, especially in water and PBS. CAPA films showed improved overall stability, although alginate-rich CAPA samples still exhibited considerable mass loss. Phytic acid release profiles showed that all CAPA films released PA progressively over seven days, with chitosan-rich (5:1) and moderately alginate-rich (1:2) samples releasing the highest amounts. The lowest release was observed for the 1:5 ratio, suggesting lower PA binding in highly alginate-rich systems, as indicated by semi-quantitative EDX analysis. Release trends correlated with swelling and mass loss, indicating that the polymer ratio governs PA release and network stability. Cytotoxicity of PA-free materials did not affect the viability of MG-63 and HDFa cells. On the contrary, CAPA films were cytotoxic to both cell types at the highest concentration due to PA release, which was supported by a PA dose–response assay.

## Figures and Tables

**Figure 1:**
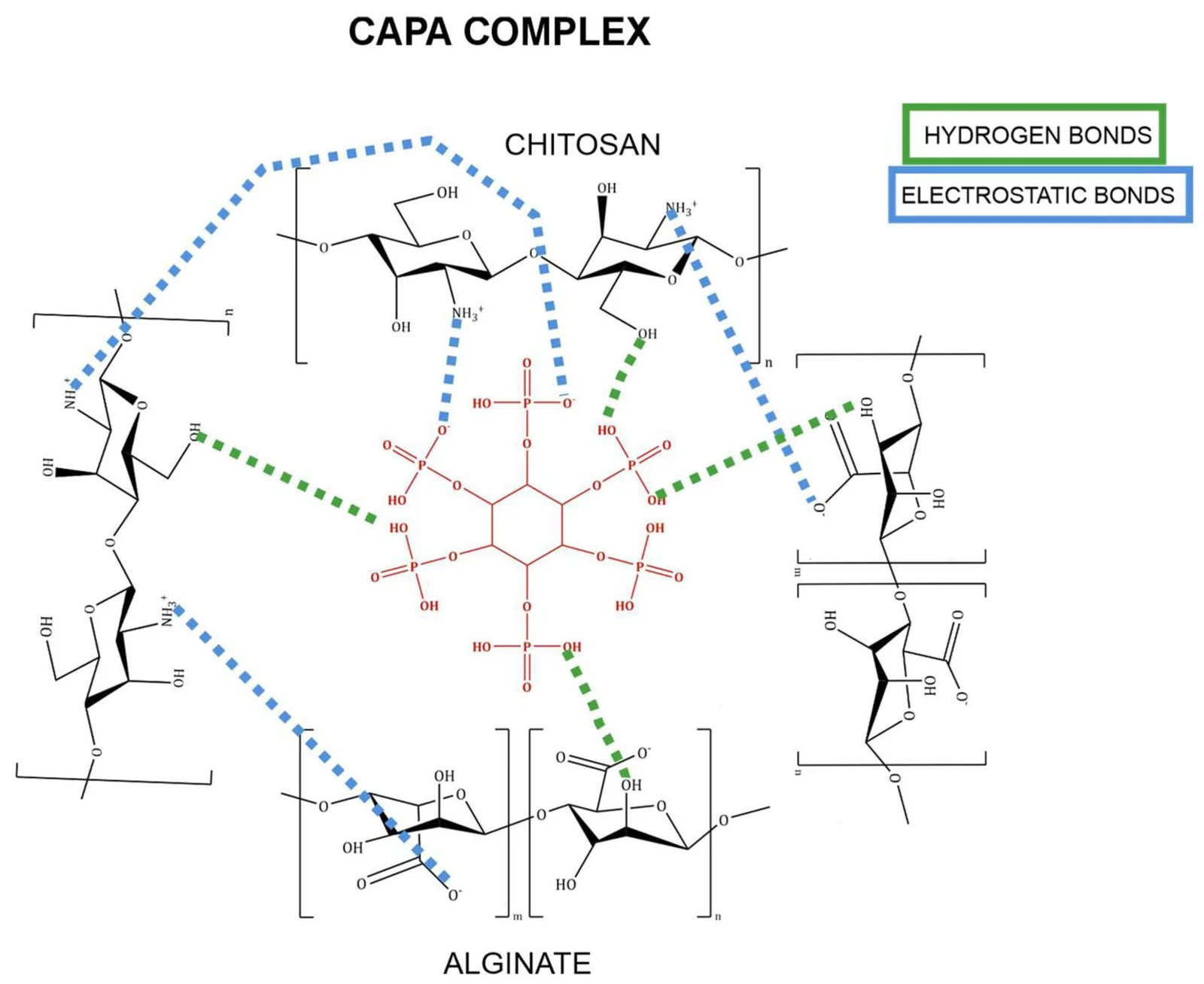
Schematic illustration of assumed interactions in PA-crosslinked PEC. Green dashed lines denote hydrogen bonding, while blue dashed lines denote electrostatic interactions.

**Figure 2:**
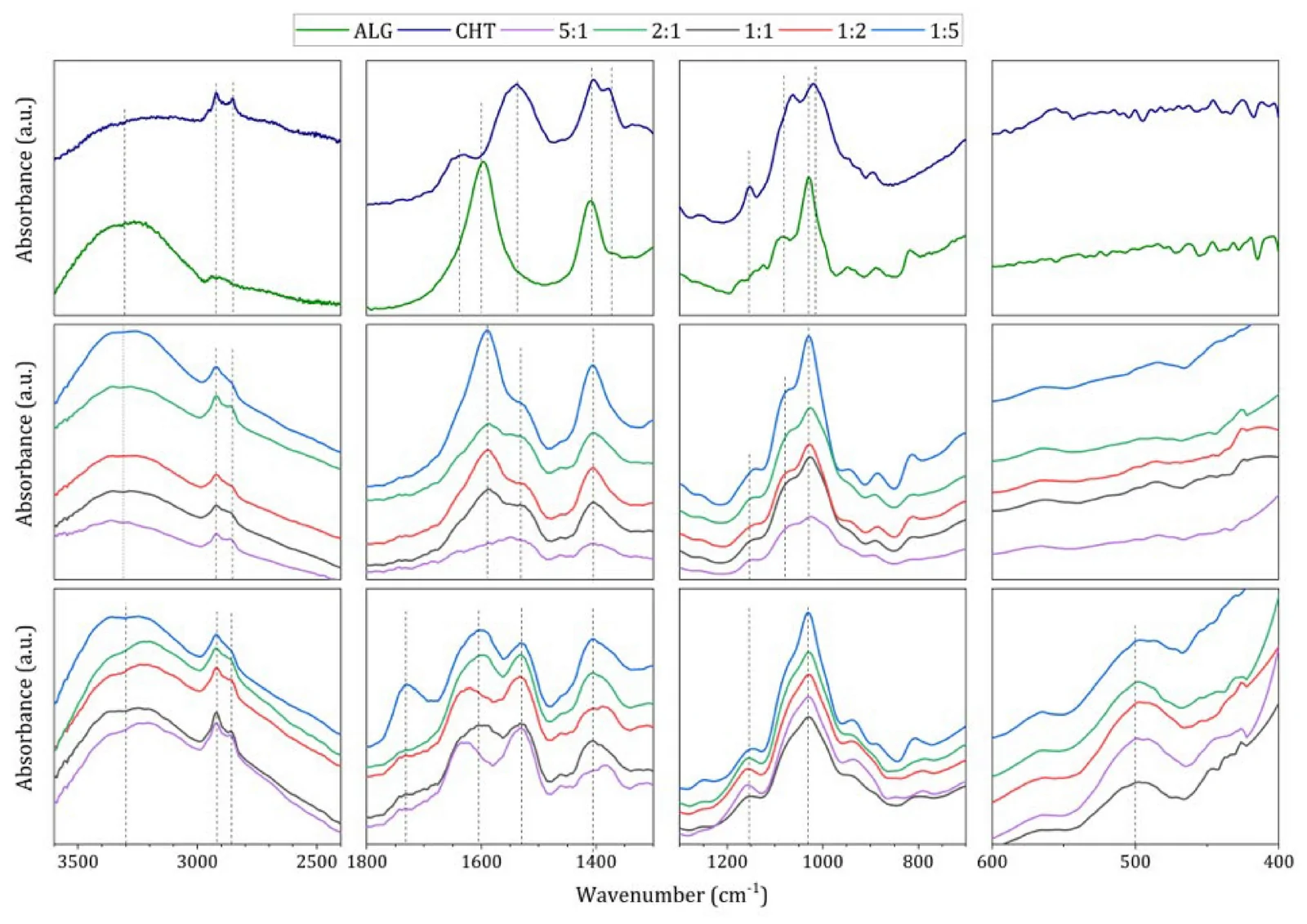
ATR-FTIR spectra, in the order of (**top**) chitosan and alginate films, (**middle**) CA films, and (**bottom**) CAPA films. The spectra were vertically shifted for clarity. Dashed lines denote the main functional groups.

**Figure 3:**
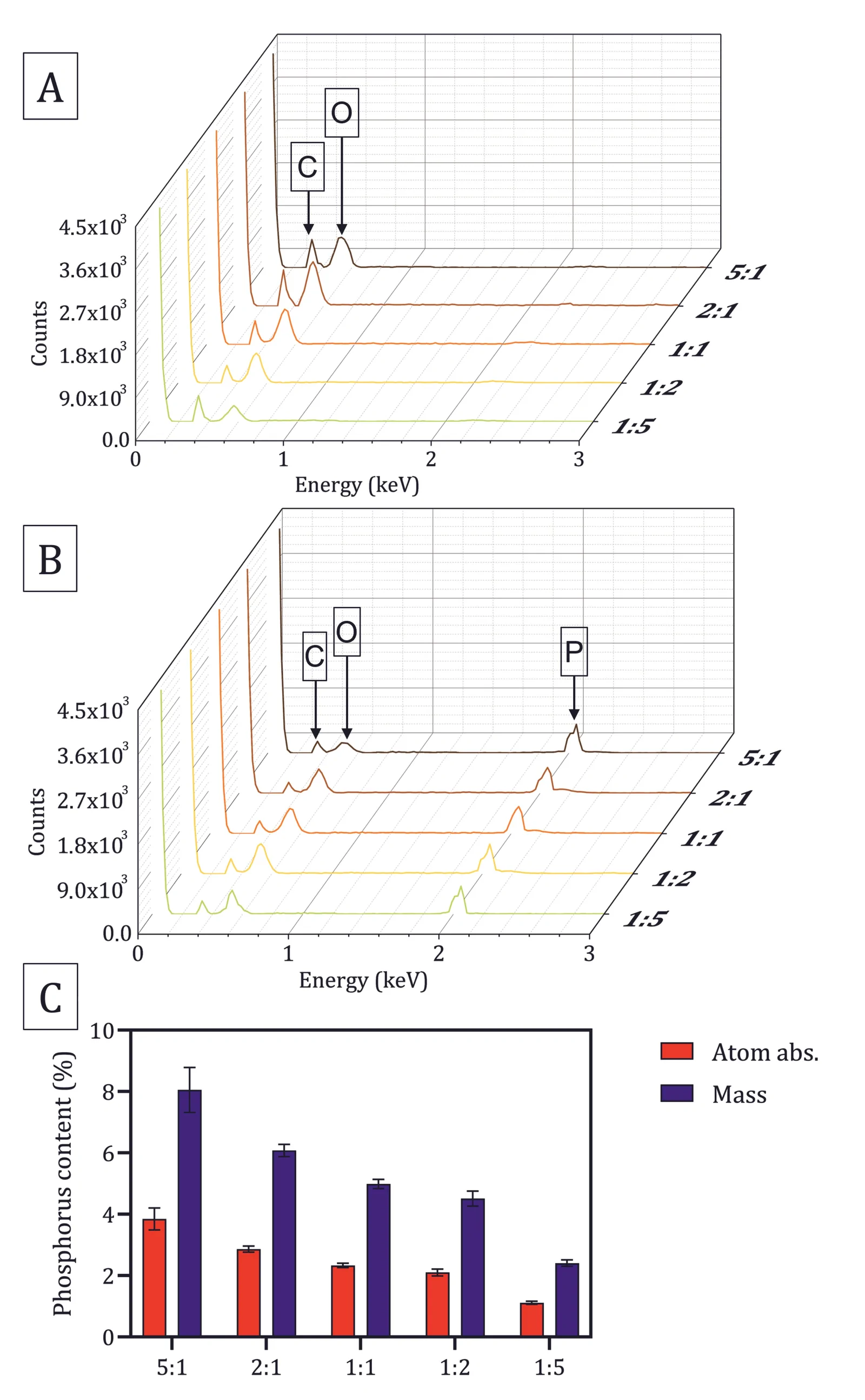
EDX spectra of (**A**) CA and (**B**) CAPA films. The phosphorus content obtained via EDX analysis (**C**).

**Figure 4:**
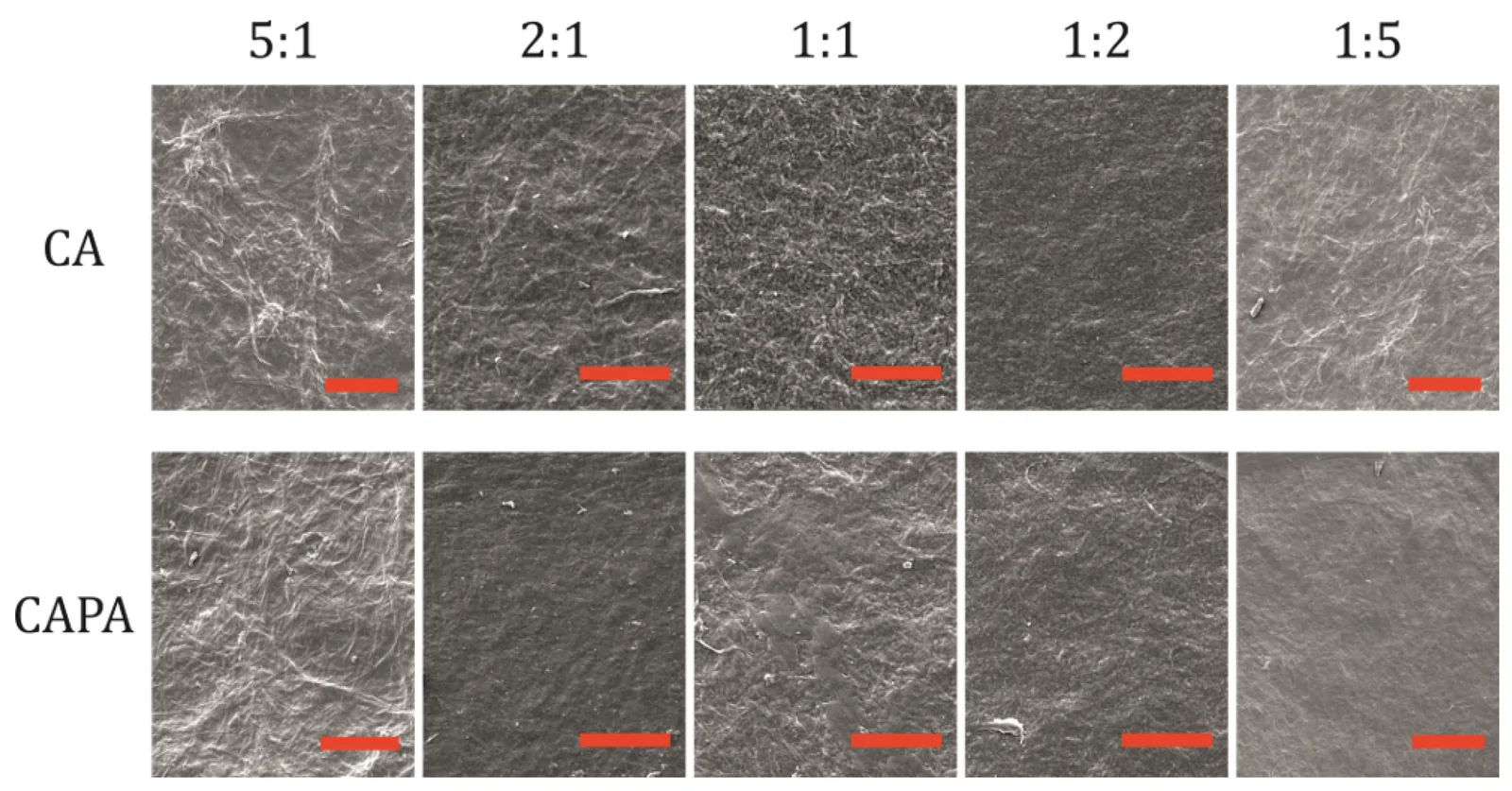
SEM images of CA and CAPA surfaces at different C:A ratios. The scale represents 50 µm.

**Figure 5:**
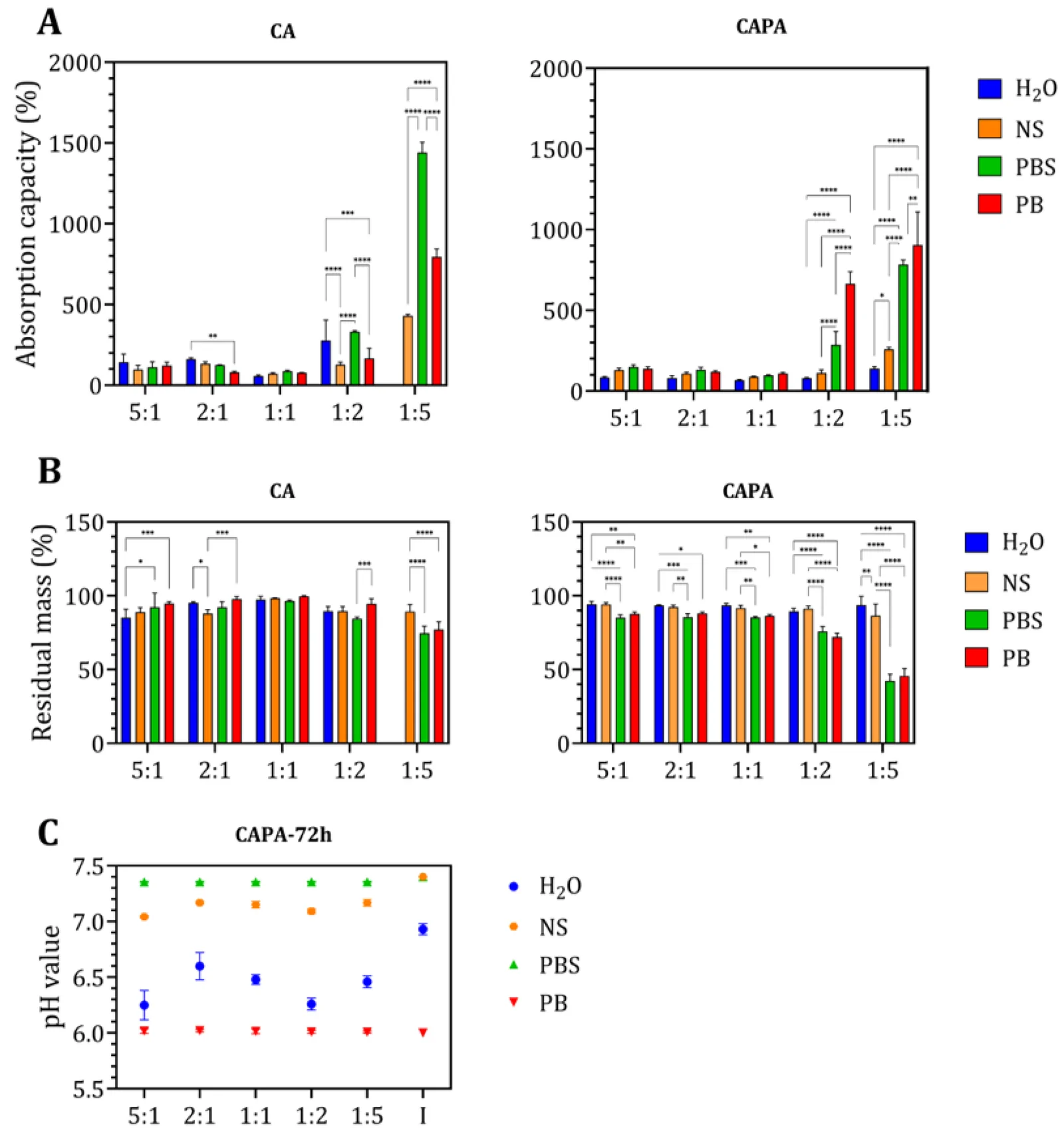
(**A**) Swelling capacity of CA and CAPA films after 72 h of immersion in different solutions. (**B**) Residual mass of CA and CAPA films after 72 h of immersion. (**C**) Changes in pH value after 72 h of immersion of CAPA films in different media, where I represents the initial pH value of the media. For the CA with a ratio of 1:5, samples were dissolved after 24 h. Results are presented as means ± standard deviation. Significant differences between groups: * *p* < 0.05, ** *p* < 0.01, *** *p* < 0.001, **** *p* < 0.0001.

**Figure 6:**
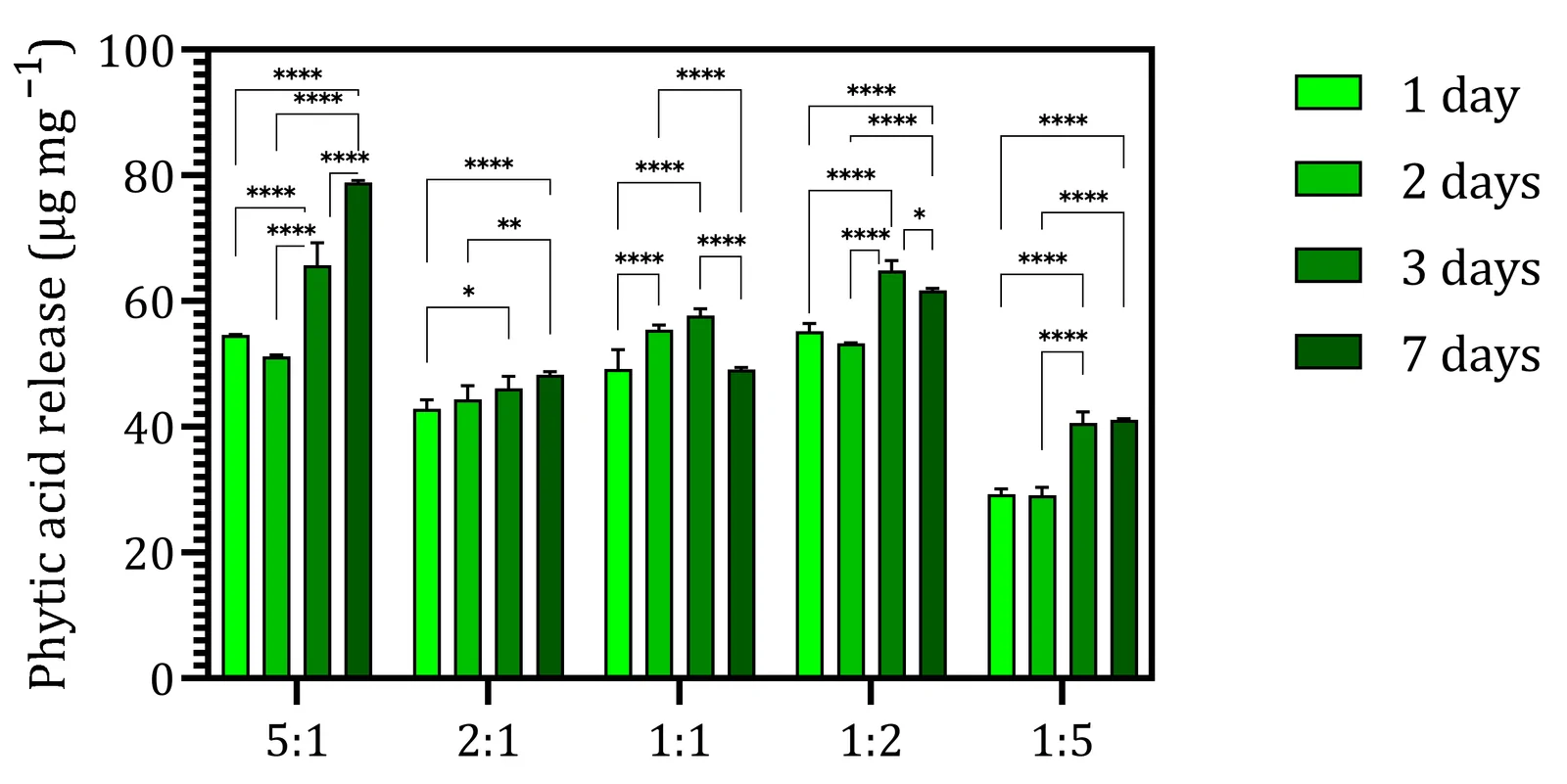
Phytic acid release profile in normal saline, pH 7.4, and at 37 °C. Release was assessed by individual measurement at each time point. Results are presented as means ± standard deviation from three independent measurements. Significant difference between groups: * *p* < 0.05, ** *p* < 0.01, and **** *p* < 0.0001.

**Figure 7:**
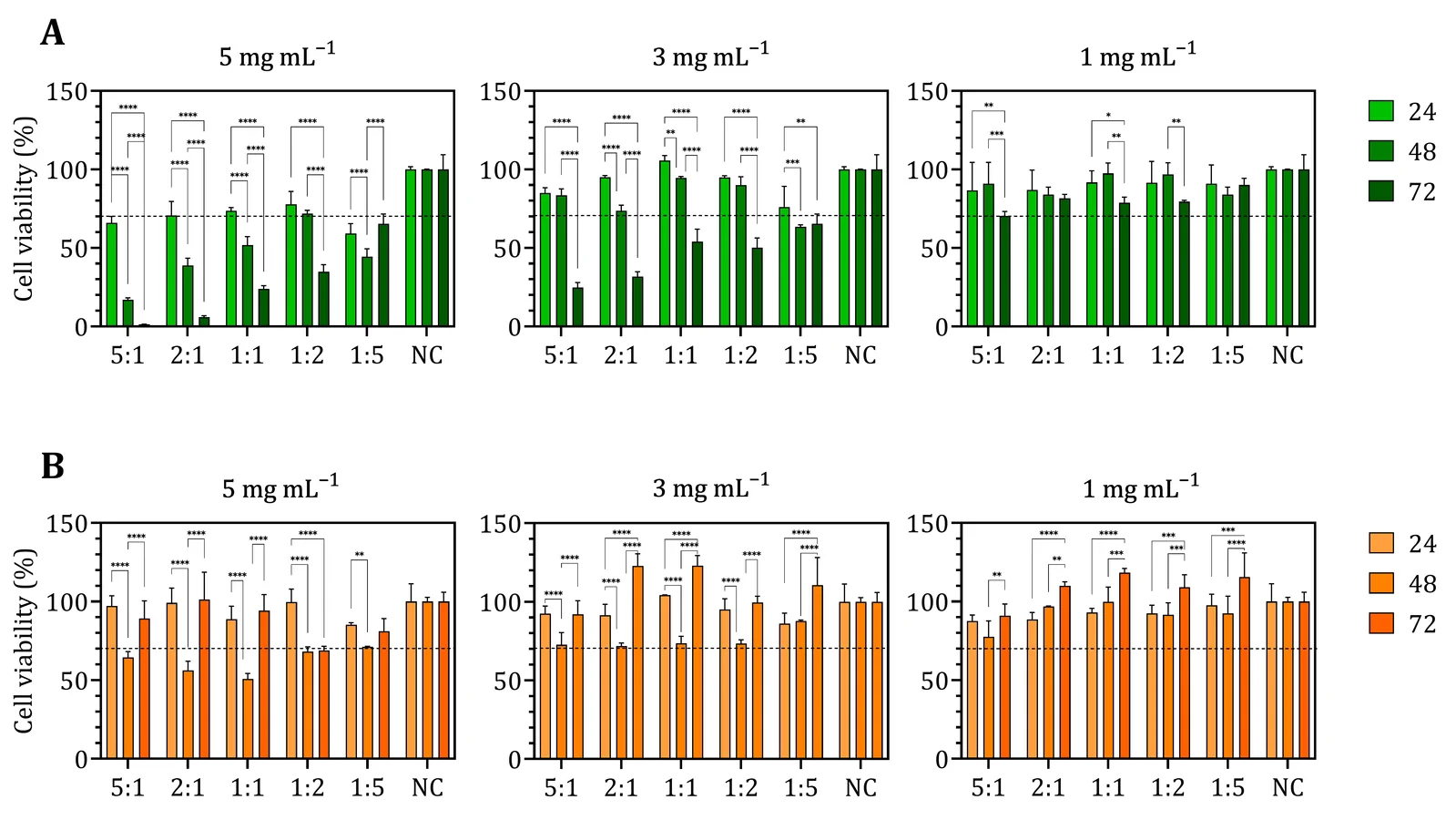
Cytotoxicity assay of CAPA materials on (**A**) MG-63 and (**B**) HDFa cells at 24, 48, and 72 h. NC indicates negative control. The cytotoxicity threshold was set at 70% cell viability (black dotted line). Values above 70% are classified as non-cytotoxic, and values below 70% as cytotoxic (ISO 10993-5:2009 [34]). Results are presented as means ± standard deviation from six independent measurements. Significant difference between groups: * *p* < 0.05, ** *p* < 0.01, *** *p* < 0.001 and **** *p* < 0.0001.

**Table 1:** Chitosan-to-alginate (CA) solution ratio for CA and CAPA films.

V (Chitosan), mL	V (Alginate), mL	Ratio C: A	Charge Ratio, n(NH_3_^+^): n(COO^−^)
25	5	5:1	5.30:1
20	10	2:1	2.10:1
15	15	1:1	1.05:1
10	20	1:2	0.52:1
5	25	1:5	0.21:1

**Figure A1:**
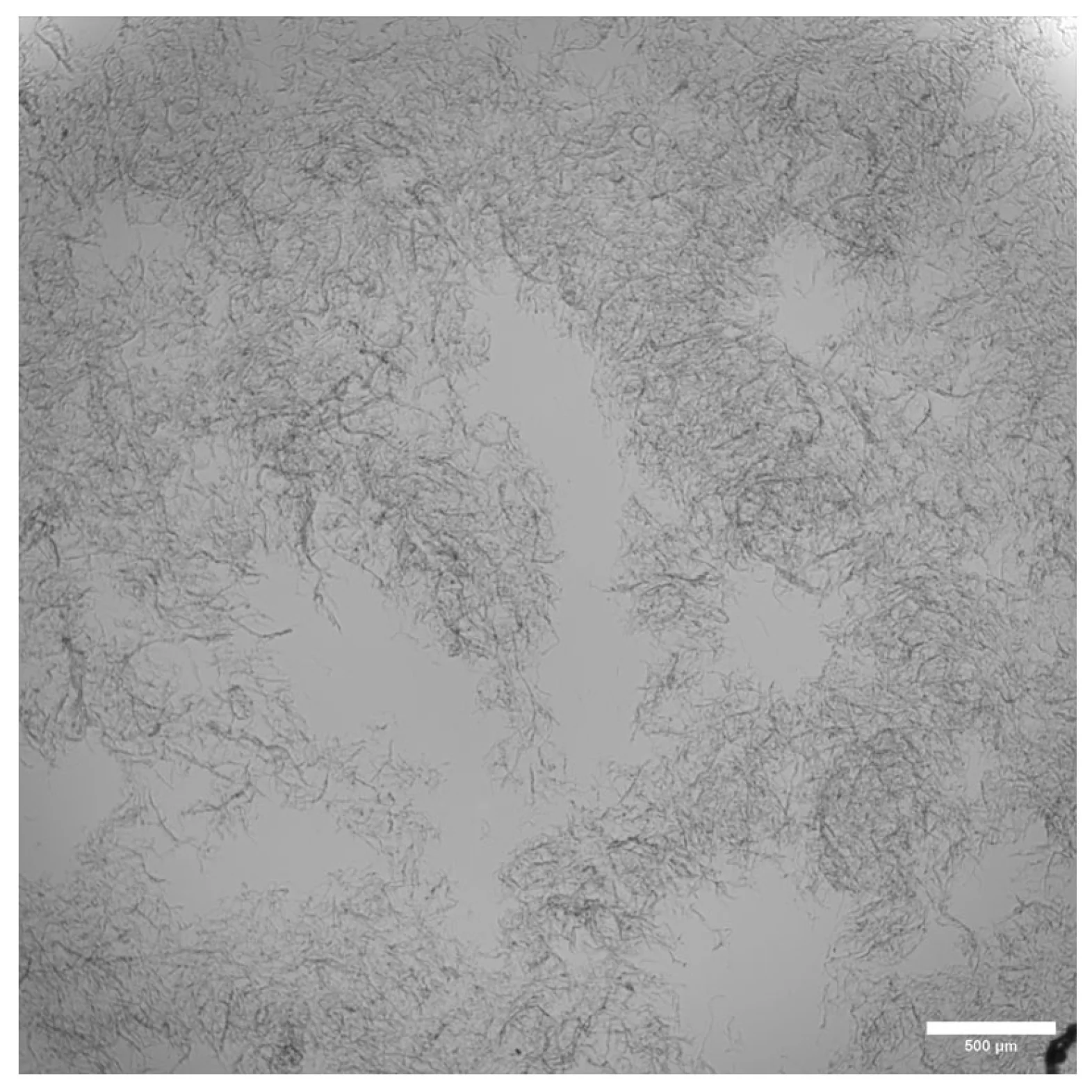
Produced CA microgels at room temperature, with a C:A ratio of 1:1. Scale bar: 500 μm.

**Figure A2:**
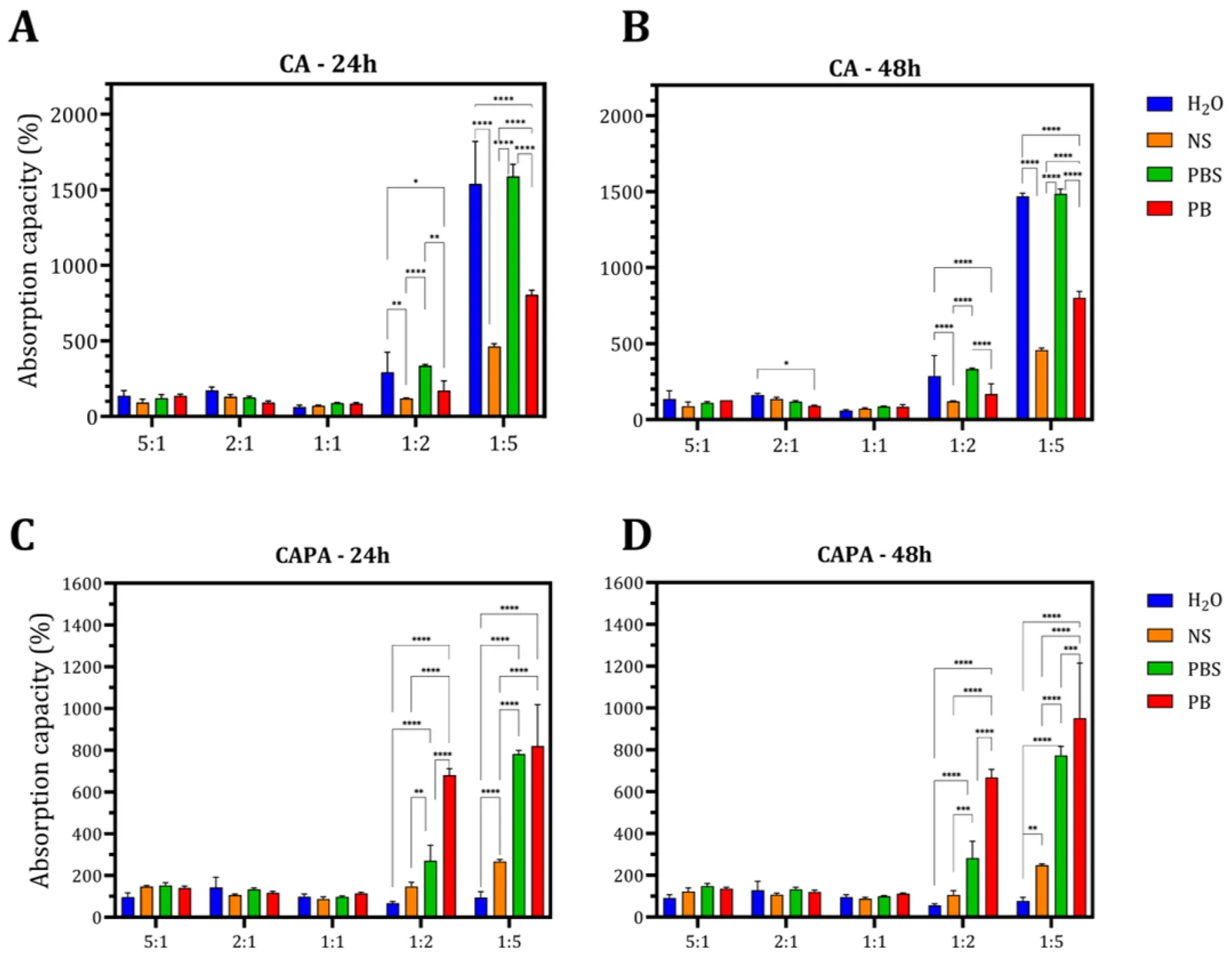
Swelling of the CA and CAPA in different media. (**A**) Swelling of CA after 24 h, (**B**) CA after 48 h, (**C**) CAPA after 24 h and (**D**) CAPA after 48 h. Results are presented as means ± standard deviation. Significant differences between groups: * *p* < 0.05, ** *p* < 0.01, *** *p* < 0.001, **** *p* < 0.0001.

**Figure A3:**
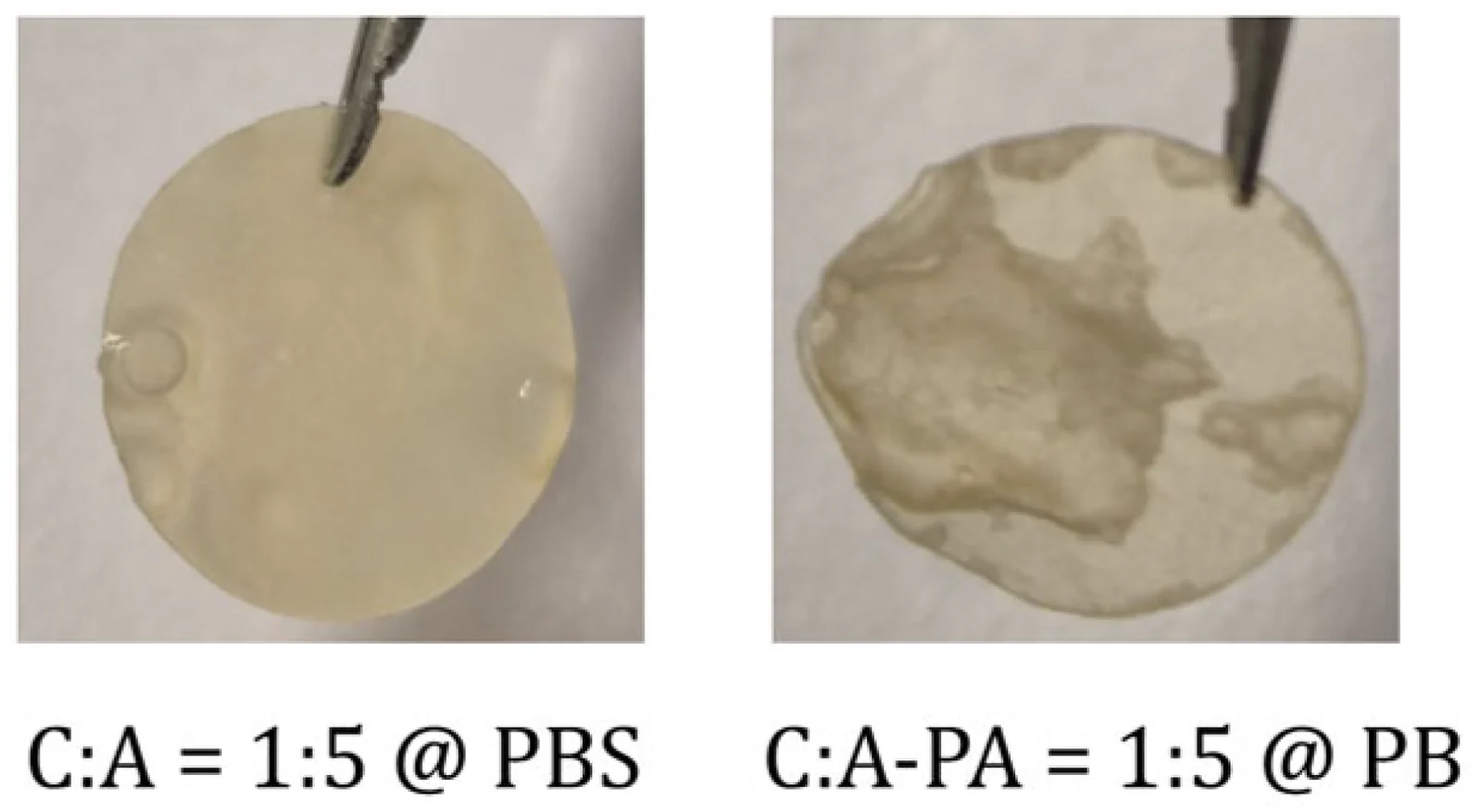
Deformation of the swollen samples occurs. Delamination of samples with excess alginate is more pronounced after crosslinking with PA. For reference, it is possible to see the control sample with a ratio of 1:5 and pockets that occur independently of crosslinking.

**Figure A4:**
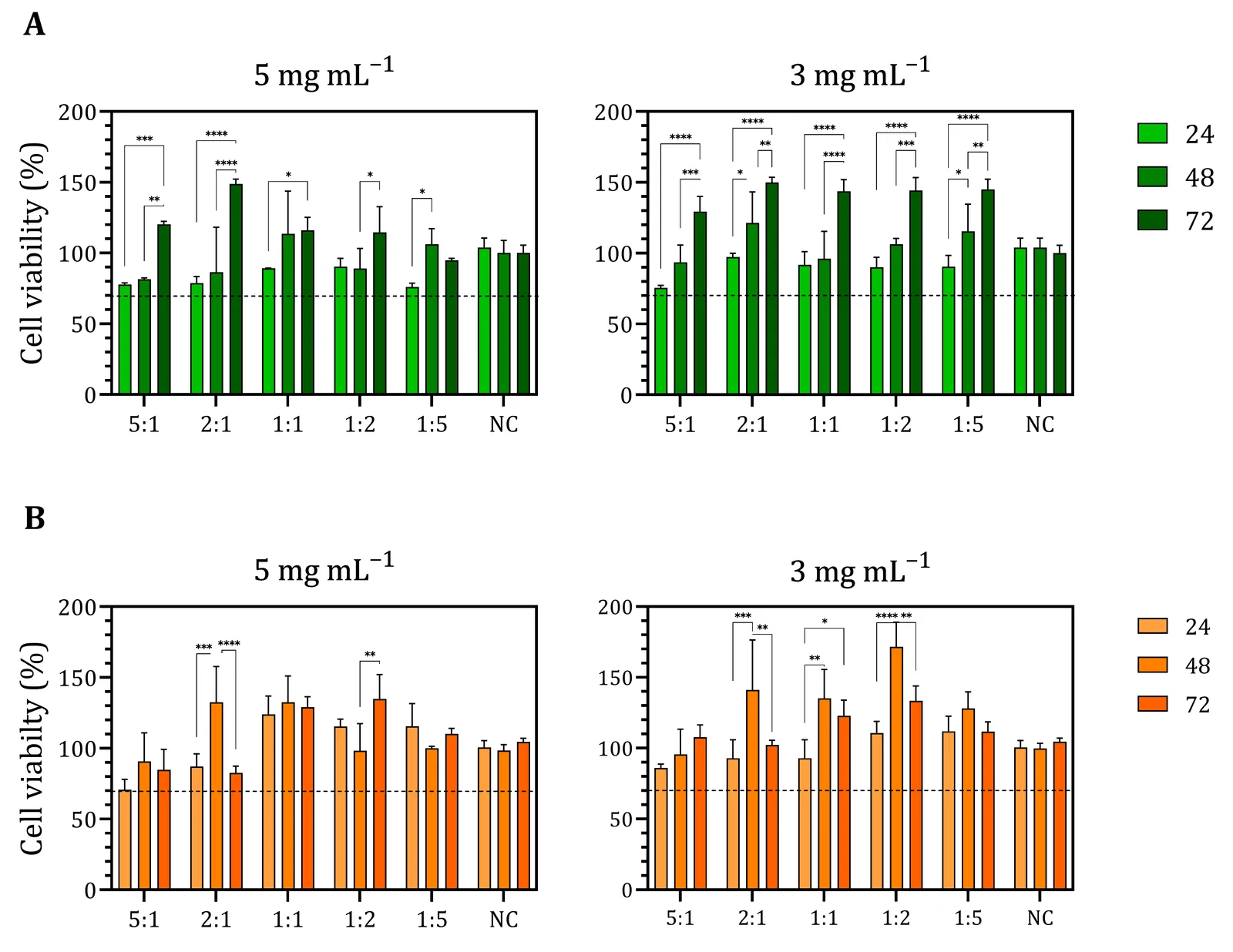
Cytotoxicity assay of CA films on (**A**) MG-63 and (**B**) HDFa cells at 24, 48, and 72 h. NC indicates negative control. The cytotoxicity threshold was set at 70% cell viability (black dotted line). Values above 70% are classified as non-cytotoxic, and values below 70% as cytotoxic (ISO 10993-5:2009 [34]). Results are presented as means ± standard deviation from three independent measurements. Significant difference between groups: * *p* < 0.05, ** *p* < 0.01, *** *p* < 0.001 and **** *p* < 0.0001.

**Figure A5:**
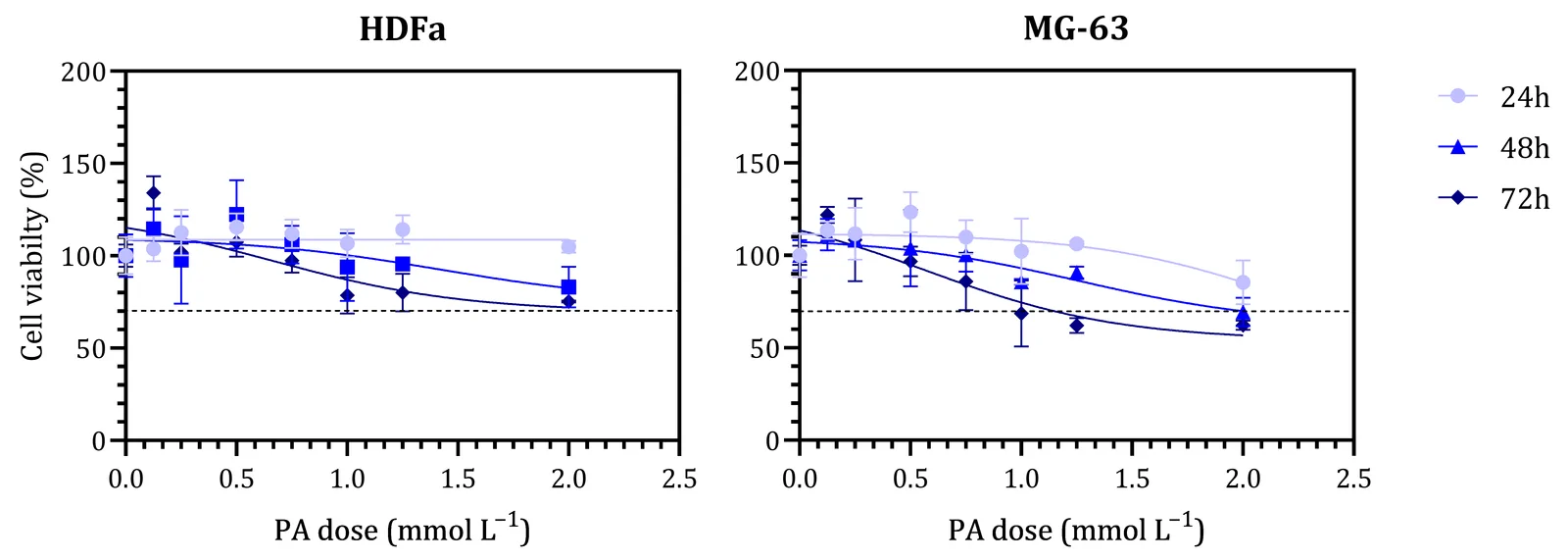
Cell viability in response to the free PA for MG-63 and HDFa cells at 24, 48, and 72 h, and IC_50_ values estimated after 72 h. The cytotoxicity threshold was set at 70% cell viability (black dotted line). Values above 70% are classified as non-cytotoxic, and values below 70% as cytotoxic (ISO 10993-5:2009 [34]). Results are presented as means ± standard deviation from three independent measurements. The three-parameter model generated stable parameter estimates (bottom = 69.58; top = 125.2; IC_50_ = 4.585 mmol L^−1^) with markedly improved fit quality (R^2^ = 0.601) for 72 h for HDFa; for MG-63, the three-parameter model yielded stable parameter estimates (bottom = 54.07; top = 129.5; IC_50_ = 3.727 mmol L^−1^) and provided the best goodness-of-fit (R^2^ = 0.711).

## Data Availability

Data will be available upon a reasonable request.

## Abbreviations

The following abbreviations are used in this manuscript:

ATR-FTIR	Attenuated Total Reflectance Fourier-Transform Infrared Spectroscopy
CAPA	Chitosan–Alginate–Phytic Acid
HDFa	Human Dermal Fibroblasts
HPDL	Human Periodontal Ligament Fibroblasts
EDX	Energy-dispersive X-ray Spectroscopy
MTT	((3-(4,5-dimethylthiazol-2-yl)-2,5-diphenyltetrazolium bromide) assay
PBS	Phosphate-buffered Saline
PEC	Polyelectrolyte Complex
SEM	Scanning Electron Microscopy
CA	Chitosan–Alginate
NC	Negative Control
NS	Normal Saline
PA	Phytic Acid
PB	Phosphate Buffer
SI	Selectivity Index
